# Toxic Release Damage Distance Assessment Based on the Short-Cut Method: A Case Study for the Transport of Chlorine and Hydrochloric Acid in Densely Urbanized Areas in the Mediterranean Region

**DOI:** 10.1021/acs.chas.2c00095

**Published:** 2023-07-12

**Authors:** Angela M. Tomasoni, Abdellatif Soussi, Roberto Sacile

**Affiliations:** Department of Informatics, Bioengineering, Robotics and Systems Engineering, University of Genoa, 16145 Genoa, Italy

**Keywords:** safety transport, dangerous goods, ICT data monitoring, chlorine scenario, decision support system, risk evaluation

## Abstract

The transportation of dangerous goods by road is the most accident-prone mode of transportation, even if accidents involving road transportation of dangerous goods are considered as a Low Probability and High Consequence event (LPHC event). However, several dangerous goods are transported by road networks, such as petroleum products and chemicals, which can generate major dangerous consequences such as spills, explosions, fires, or toxic clouds. In this context, this article presents a method to calculate and quickly quantify the sizes of impact zones characterized by high lethality and irreversible injuries to people in the case of a hazardous materials transport accident. This method is used as a module for the analysis of the consequences of different potential accident scenarios, for the Web-GIS platform proposed by LOSE+LAB, that implements appropriate ICT tools and systems for monitoring the flow of goods that would enable a continuous monitoring system at the cross-border level and transmit data and information to the territory actors involved in the management of dangerous goods according to the ADR standard. The proposed method provides the user with a visualization of the possible outcomes of an event by reproducing the impact area for different accident scenarios, which can provide quick maps of the hazard and represents a decision support system for territorial governance in terms of intervention and response protocols for emergency management in the cases of dangerous goods accidents.

## Introduction

Dangerous products are defined as chemicals and pesticides (materials or substances), which, due to their physical, chemical (toxicity, reactivity, etc.), and physiological characteristics, may present risks for humans, property, infrastructure, and/or the environment.^1^ The main consequences of an accident during Dangerous Goods Transportation (DGT) may involve fire, explosion, release of a toxic cloud, and air-dispersion, soil, and/or water pollution.^2^ These accidents are mainly caused by corrosion, mechanical failure, operational/human error, natural hazards, and equipment failure.^3^ A toxic cloud release^4^ can be due to a toxic product leakage or dispersion following an explosion, and the dispersion of combustion products following a fire of harmful chemicals (even if the initial product is nontoxic).^5^ This cloud will move away from the accident site according to the winds that are active at the time and can affect people located at great distances from the initial source point, despite the obstacles and natural and urban barriers involved.^6^

For this reason, starting from the approach that the observed knowledges are at the bases of “planning” and “doing” in case of a pollutant accident involving people—“using” a decision support system (DSS) as shown in ref (7)—it is possible to give an appropriate suggestion and some operative advice (qualitative information, time steps, and areas of involvement) in order to “manage” a potential risk. The DSS should be based on potential hazard identification, using simplified tools, different from the 3D multiscale weather forecast Atmospheric Transport and Dispersion models embedded into the DSS.^8^ Such a DSS may be also useful for teaching and training with regards to the basic hazard consciousness of operators, and to assess the potential hazard of the new energy vectors^9^ or describing another HazMat means of transport to generate risk maps by superimposing the hazard and vulnerability using GIS software.^10^

The present research adopts a bottom-up approach, emphasizing the importance of having the necessary knowledge, tools, and numerical and physical resources available at the time and place of an accident to effectively address the associated risks and minimize the potential consequences. Accordingly, the research begins with the real-time collection of relevant data in a specific case study area, with the goal of progressively refining hazard quantification over time.

Several modeling methods and software tools have been developed to determine the impact areas in case of accidents involving **Haz**ardous **Mat**erial (HazMat)^11−13^ (e.g., Phast,^14^ Riskcurves,^15^ HAMS-GPS,^16^ and ALOHA^17^). These methods and tools assist decision-makers and authorities in setting up emergency management response plans and protocols,^18^ and/or HazMat flow planning according to the urban mobility, as part of the planning process to reduce risk and increase safety for people living there in/along the territory involved.

As part of the LOSE+ project,^19^ a Web-GIS platform has been implemented to represent a decision support system for territorial governance in the event of an accident. The platform aims to increase the level of knowledge about the flows of HazMat in the ports of Genoa (Liguria Region, Italy), Toulon (Var department, France), Capraia Island, Livorno, Piombino, Portoferraio (Tuscany Region, Italy), Olbia, and also Porto Torres port (Sardinia Region, Italy).^20^ For the sake of brevity, we will describe only two case studies of the entire territory considered.

**Figure 1:**
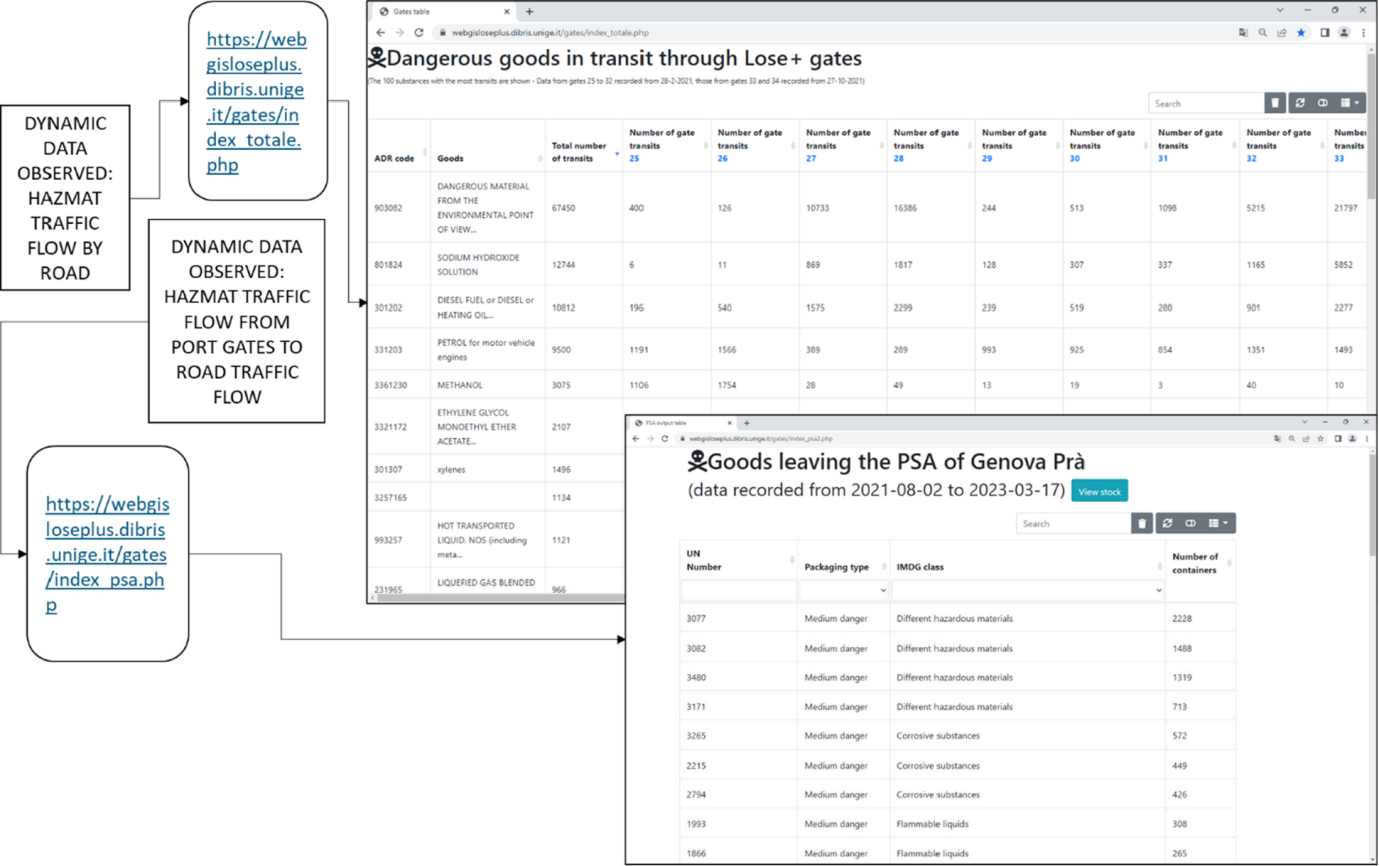
Description of the LOSE+ platform data collection process.

This platform takes advantage of innovative technologies to increase safety on the road network by installing a camera system on the main traffic routes in the study areas, along the coastal area, near the port crossings, as well as in the territories observed by the LOSE+ project, as a common safety system at the Mediterranean scale. These tools enable the local public authority to efficiently manage traffic, with an accurate and timely view of the vehicle situation. In addition, the captured images are processed to identify vehicle classification and recognize dangerous goods license plates according to the ADR standard^1^ and IMDG Code^21^ (Figure 1).

In the LOSE+ system, monitoring data has been available for the last two years. The transit frequency of marine pollutant HazMat is higher than all the other classes of substances, with 5.748 containers. The transport of toxic materials is represented by 1003 containers leaving the port to be transported by road and train. Chlorine and hydrochloric acid are included in this set of data and class of HazMat, which is described further in the case study section.

The information received and collected in the platform database allows public and private operators to efficiently manage the hazard and potential risks related to the DGT on Italian and French territory. A simulation model of different HazMat scenarios in case of an accident is also available to assist in managing these risks.

The proposed model for this case study, based on the Short-Cut Method, enables public authorities and decision-makers to determine the impact area in the case of an accident involving dangerous goods in transit in the ports of Genoa (Liguria Region) and Olbia (Sardinia Region). The model provides the user with a visualization of the possible outcomes of an event by drawing the impact areas for different accident scenarios, which can provide quick maps of the hazard near the selected area.

## Methodology

The Short-Cut Method^20^ is an expedited approach that can be used to estimate damage distances resulting from incidents involving releases of hazardous substances from various types of containers, including those stored in confined containers, and/or those transported by ship, tanker truck, tank train, and pipeline (the latter types are excluded from the scope of Legislative Decree 334/99). The method classifies flammable and toxic substances according to their generally significant hazard characteristics to evaluate their potential consequences. For each risk class, the method provides an indication of the accident scenarios with the highest and medium probability of occurrence (typical hazardous material accident outcomes can be named differently depending on the thermodynamic–chemical–physical phenomenon that is developed: pool fire, flash fire, Vapor Cloud Explosion (VCE), or toxic cloud).

The distances containing the possible consequences (exposed elements and damage) are given in a table format, according to the classes of HazMat, the different quantities of product, the four lethality thresholds, and the two categories of weather conditions according to the Pasquill classification (D5 and F2).^22^

The Pasquill stability classes are used to estimate the atmospheric dispersion of pollutants from a release point. Pasquill class D5 represents “very stable” atmospheric conditions, which occur at night or during the early morning when the ground is colder than the air above it and little mixing occurs. This results in a very limited dispersion of hazardous materials.

Pasquill class F2, on the other hand, represents “moderately unstable” atmospheric conditions, which occur during the daytime when there is a greater likelihood of mixing due to solar heating. This can result in more significant dispersion of hazardous materials.

In length, these obtained distances represent the radius of a circular area that approximately corresponds to the potential impact area of the accidental event considered.

The application of the Short-Cut Method involves the following steps:

Step 0: Select the relevant substance for the risk assessment.

Step 1: Check if the selected substance is on the substance list for the Short-Cut Method.

Step 2: Retrieve the list of Short-Cut classes associated with the substance.

*-If the substance is associated with the classes of flammable liquids or flammable gases*: the user must select the characteristics of the container to identify the specific hazard class in the Short-Cut Method. The reference table is then used to determine the radius of the impact area.

*-If the substance is associated with toxic substance classes*: apply the Paradigm procedure and define the four values A, B, C, D as follows:

1. Identification of the value “Reference numbers (ref.)” depends on the substance and the type of containment. Reference numbers include Class 4 Toxic liquids; Class 5.1 Toxic gases liquefied by compression; Class 5.2 Toxic gases liquefied by refrigeration; Class 5.3 Toxic gases simply compressed.
  a. Identification of the A value, which corresponds to the type of detention and is identified by consulting Table 1.
  b. Identification of the B value through Table 2, which corresponds to the quantity of goods stored. This step helps to estimate the amount of the substance that is present, which is important in assessing the severity of the risk.
  c. Identification of the C value through Table 3, which represents the lethality threshold of the substance. This value is critical in determining the potential harm to human health and the environment and helps to guide decisions about emergency response.
  d. Finally, identification of the D value through Table 4, which represents the weather parameter. This step is important because the weather can significantly affect the dispersion of hazardous materials and can impact the radius of the impact area.
  By following these steps, users can effectively apply the risk assessment method and identify the appropriate radius of the impact area in the event of an accident involving hazardous materials.
2. Using Paradigm, search for tables assigning individual substances to classes.
3. Identification of the radius of the impact area by consulting the relevant table in Appendix 1 of the Short-Cut Method.^20^

**Table 1:**
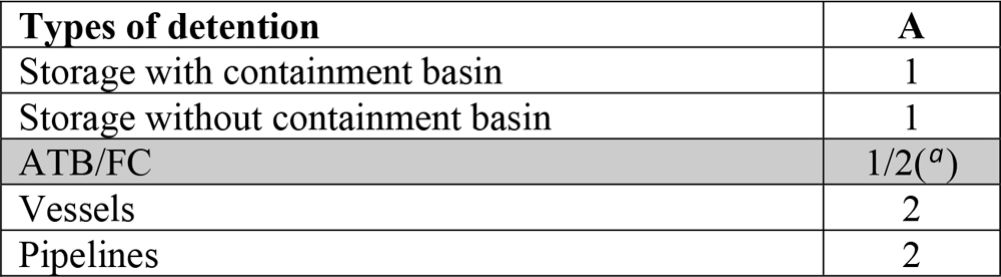
Parameter A of the Paradigm Procedure

a Must be taken as 2 if the substance falls into class 5.3.

**Table 2:** Parameter B-1 of the Paradigm Procedure for the Type of Transport via ATB/FC

	**B**	
**ATB/FC**	**Most probable hypothesis**	**Average hypothesis**
Toxic liquids	47	47
Toxic gases (all physical phases)	48	48

**Table 3:** Paradigm Procedure Parameter C

**Threshold**	**C**
LC50	1
IDLH	3

**Table 4:** Paradigm Procedure Parameter D

**Weather**	**D**
D5	1
F2	2

We note that for certain substances such as hydrochloric acid, hydrofluoric acid, and nitrogen monoxide, there may be a lack of qualified data in DIPPR (Design Institute for Physical Properties). However, these substances are still included in the respective class as they can be classified based on the established criterion. The classification and corresponding parameters for these substances are directly defined according to the table provided in the SCM’s appendix.

In the Short-Cut Method, a macro-classification of toxic substances is defined based on how they are held or formed, as shown in the Table 5.

**Table 5:** Macro-classification of Toxic Substances According to the Short-Cut Method

**Macro-classes**	**Type of substance**		**Additional features**
4	Toxic liquids		-
5.1	Flammable gases	liquefied by compression	-
5.2		liquefied for refrigeration	-
5.3		compressed	-
6	Toxic products of combustion	from pesticides	dioxin precursors
			dioxin precursors
		from fertilizers	from nitrogen fertilizers
			from sulfur fertilizers
		from plastics	-

In this case study, we considered the tanker transport case (ATB) for the two selected scenarios.

## Case Studies

The effects of an accidental event involving HazMat affect the impact area with a severity generally decreasing with the distance to the point of origin of the event, except for the possible presence of a domino effect. The substances considered for the analysis have been chosen among the toxic gases and materials most transported, taking into account that—according to the data collected on the LOSE+ platform, as shown in Figure 1, and the Eurostat data on DGT—flammable liquids and gases are among the most transported substances in the EU and in Italy.^23^

The proposed method considers several transport characteristics, including the substances transported, means of transport, type of container, road conditions, and weather condition, in the geographical areas involved in the transport.

Starting from the most common transport types for the HazMat examined and the expected typical ruptures (size of rupture and duration of release), source terms were identified to be introduced into the simulation model for the two reference meteorological conditions (F2 and D5). All events are traced back to leakage and subsequent release of HazMat into the surrounding environment.

For the purpose of applying the Short-Cut Method (SCM), the damage is related to the physical effect through the vulnerability criterion, which is represented by the exceeding of a threshold value. In analogy with the provisions of the current legislation on the subject (Ministerial Decree of 09/05/2001),^24^ we refer to the four threshold values corresponding to

1: effects of high lethality (This level is associated with injuries or lethality that result in a high probability of death or severe, long-lasting health effects);
2: effects of early lethality (This level is associated with injuries that are reversible or result in a low probability of death or severe, long-lasting health effects);
3: effects involving serious irreversible injuries;
4: effects involving reversible injury.

In this study, we present an example of how the SCM method can be applied to two accident scenarios involving substances belonging to the toxic gas class. Specifically, the study demonstrates the direct use of the SCM method to obtain results for the damaged areas caused in the case of hydrochloric acid, while the Paradigm process is used to obtain results for chlorine. Moreover, two study areas with distinct properties were selected to showcase the versatility of the SCM method across different settings.

The SCM was created, in particular using GIS tools, after defining the distances related to the different threshold values, and each distance was indicated by means of a buffer around the entire extent of the roadway considered. Specifically, for both the port of Genoa and Port of Olbia, only the road section close to the port area was considered. Then, in order to highlight the exposure of the resident population in the event of an accident, an overlap was made between the risk distances highlighted by buffers and the census sections related to the 2011 population database of which the population density (people/km^2^) was considered.^25^

### Event and Effects: Case Study in Genoa

In this case study, the transport accident for 200 tons of liquefied chlorine by compression has been simulated. For an accident of relatively high probability and low magnitude (most probable hypothesis), we will identify the damage radius corresponding to the irreversible injuries (IDLH) under the meteorological conditions D5.

The SCM shows that chlorine is a toxic gas belonging to classes 5.1, 5.2, and 5.3 (toxic gases liquefied by compression, refrigeration, or compressed). The results for chlorine are from simulations, and the reference chlorine number for further treatment is shown in Table 6.

**Table 6:**
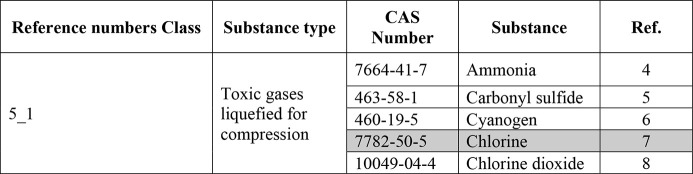
List of Substances Sorted by Class

As we demonstrate in the previous section, we proceed to identify the four numerical values (A, B, C, and D) of the Paradigm procedure using the Tables Tables 1–4.

In this case, based on the chosen mode of transport being road transport, the value of A would be 1. Additionally, for the IDLH value, the 3 corresponds to the value of C. As for the weather conditions, if the value of D5 represents the condition of the weather, then the value of D would be 1.

The identification of the value of B depends on the transported quantity of chlorine; for our scenario the value of B is 19, which corresponds to the quantity of 200 t of chlorine (Table 7).

**Table 7:**
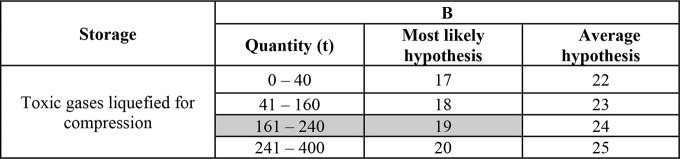
Values of B-2 According to the Amount of Chlorine Transported

The value of the required damage radius is obtained from the SCM result table for subclass 5.1.5. In this case, the damage distance is equal to 2200 m (Table 8).

**Table 8:**
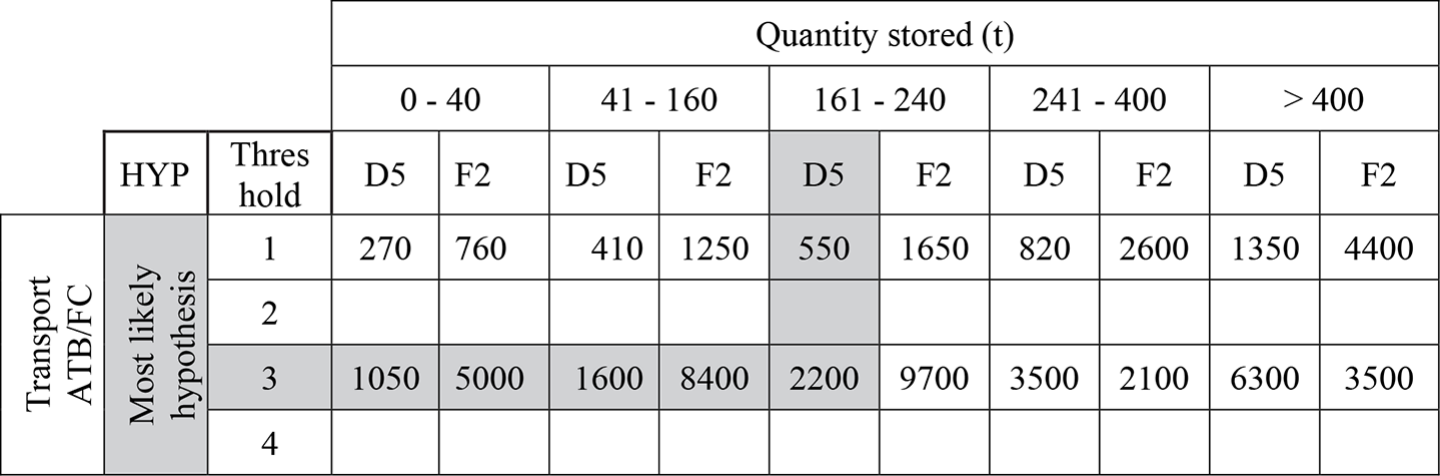
Value of the Required Damage Radius (Meters) Based on the Short-Cut Results for Subclass 5.1.5 of Toxic Gases Liquefied for Compression

**Figure 2:**
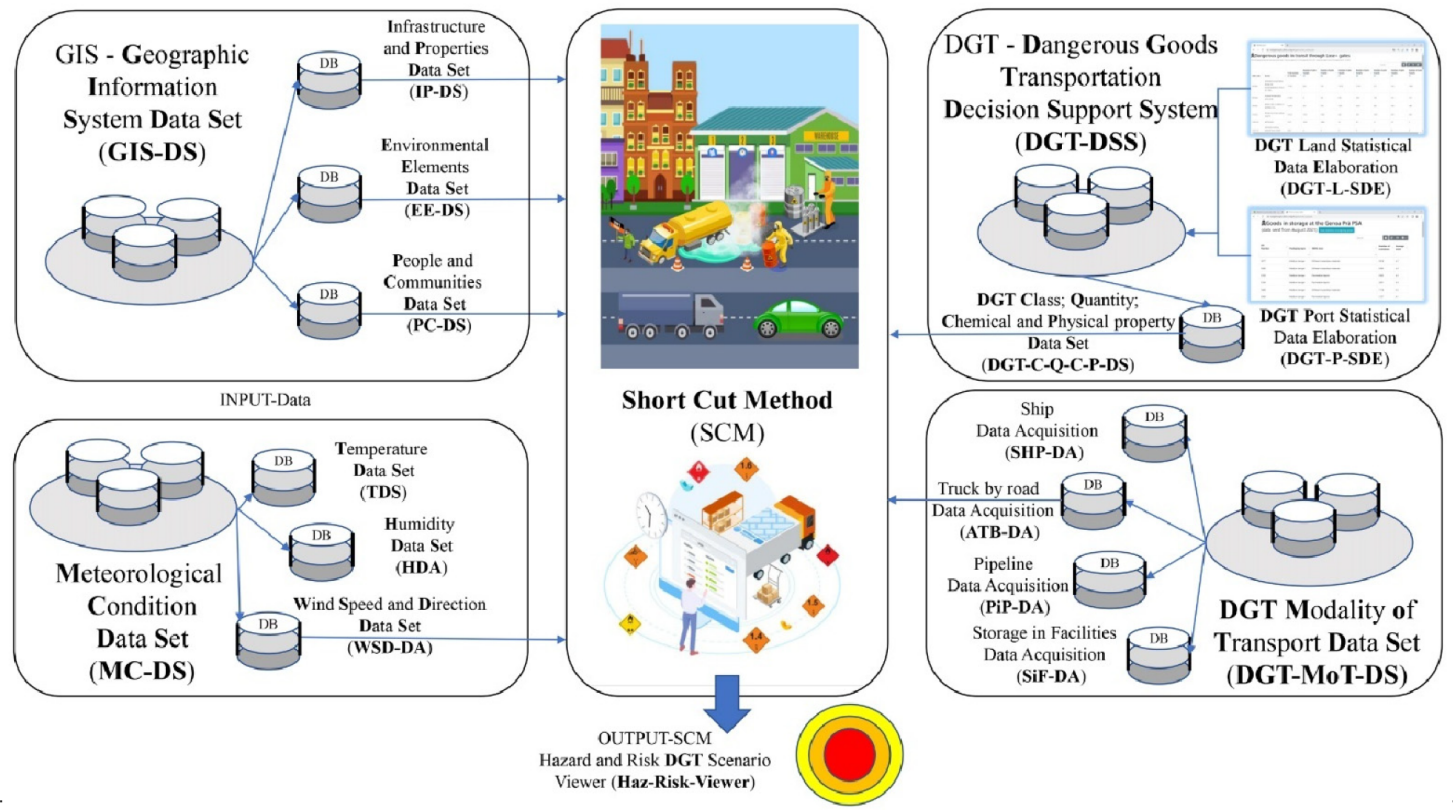
General big data architecture describing what kind of data flow is useful to generate the Hazardous and Risk Dangerous Goods Transportation Scenario.

The following image, Figure 2, shows the input data flow useful for applying the Short-Cut methodology, which gives as output data distances of damage in terms of lethal area (red area), impact area (orange area), and attention area (yellow area).

The inputs for the modeling in this case study include the type and quantity of hazardous material, means of transport, type of container, weather conditions, and population density in the areas surrounding the transport route. The outputs of the modeling are the damage radius for different threshold values and the exposure involvement of the resident population in the event of an accident, as shown in Figure 2.

The modeling tools used in this study included a platform based on GIS (Geographic Information System) technology, which was used to generate maps for each study area. The GIS platform allows for the integration and analysis of spatial data, such as transport routes, population density, and environmental conditions, to support the modeling and assessment of potential risks associated with hazardous materials transportation.

The simulated chlorine accident scenario in the urbanized area of the municipality of Genoa considered as exposed elements not only inhabitants but also schools of the Genoa district. The results of the Genoa case study scenario are shown in Table 9.

**Table 9:** Number of Residents Affected, and Schools Involved, Based on Distances at Risk Are Determined According to the Short-Cut Method

**Hypothesis**	**Weather**	**Threshold**	**Distance (m)**	**Residents urban case**	**Schools urban case**
Most likely hypothesis	D5	1: High lethality	-	-	
		2: Early lethality	-	-	
		3: Irreversible injuries	2200	114659	125
		4: Ireversible injury	-	-	

### Event and Effects: Case Study in Sardinia

In this case, the SCM is used to determine the impact area of hydrochloric acid, which belongs to the class of gases liquefied by compression. Based on the physical properties of this substance and by referring to the tables that encompass all the substances included in the method, the damage distance is determined using the table presented in Appendix 1 of the SCM. For transportation via tanker truck, the relevant reference table is Table 10.

**Table 10:**
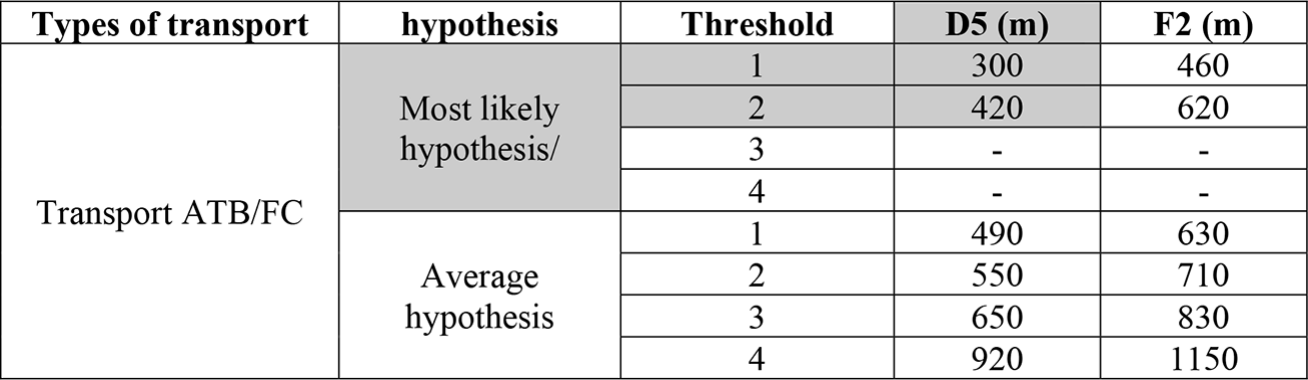
Distance of the Damage (m) According to the Short-Cut Method in the Case Studied

**Figure 3:**
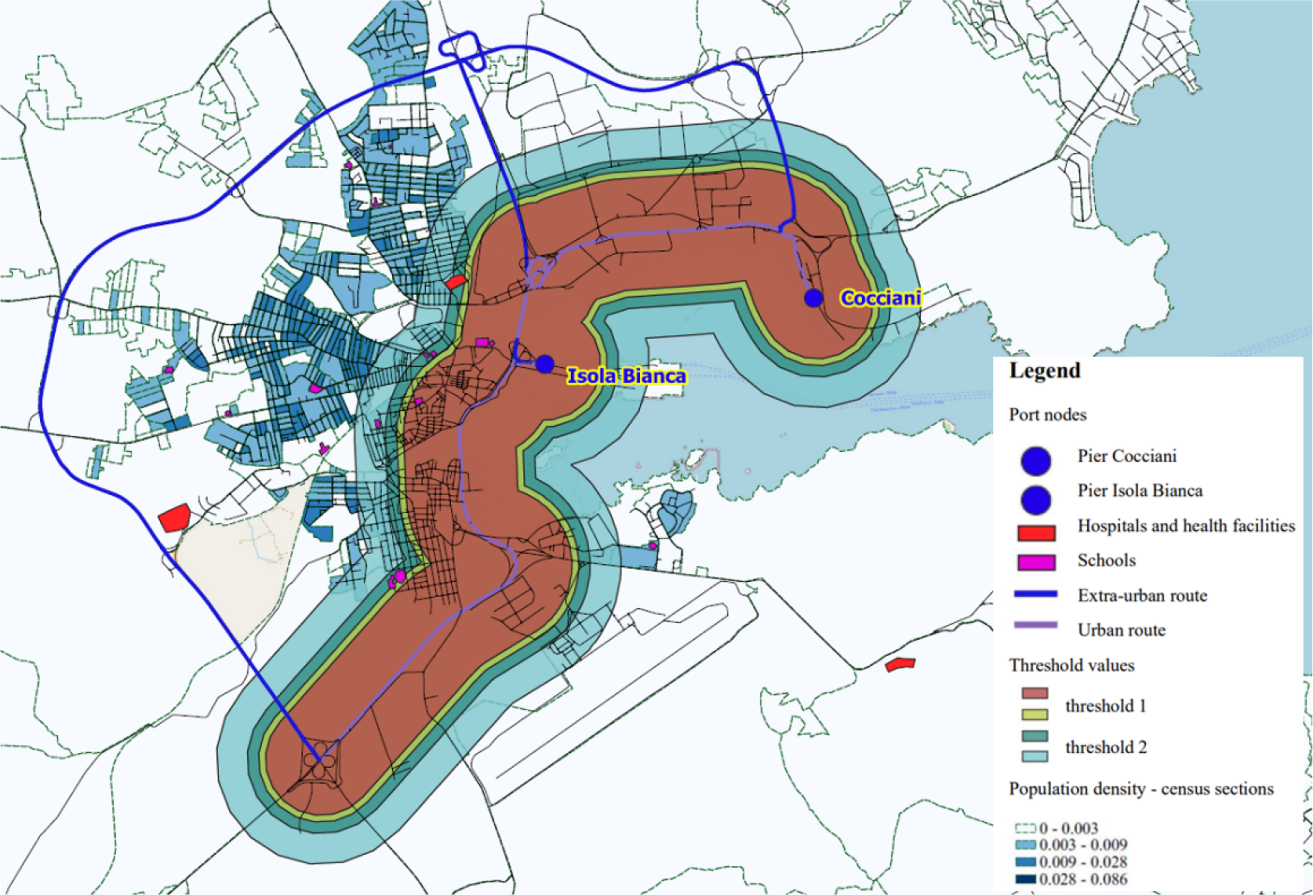
Distances for the most likely hypothesis and weather class D5—urban case. Port of Olbia.

The following image (Figure 3) represents the simulation of scenarios studied for the case of the port of Olbia using the Web-GIS software. As previously mentioned, the damage distances have been calculated with reference to two different roads that give access to the port. The distances are highlighted both in the case of the most probable hypothesis and for the meteorological condition D5. The red circles represent threshold 1 for the severe impact area derived from the assumed hydrochloric acid release scenario along a defined road infrastructure link. The yellow circles (threshold 2 in the previous table) represent the area of potential damage. Meanwhile, the green area represents the safety zone with minor potential risks.

Table 11 provides information on the residents residing in the census sections that are impacted by various damage distances under different analysis conditions. It demonstrates the number of affected residents based on the determined risk distances.

**Table 11:** Number of Residents Affected Based on Distances at Risk Determined

**Hypothesis**	**Weather**	**Threshold**	**Distance (m)**	**Residents urban case (number)**
Most likely hypothesis	D5	1: High lethality	300	2925
		2: Early lethality	420	5179
		3: Irreversible injuries	-	-
		4: Reversible injury	-	-

## Conclusion

The management of risks associated with dangerous goods transportation (DGT) involves monitoring the flows of dangerous goods (HazMat observed data) and using accident modeling and simulation techniques to understand the potential consequences generated in the different scenarios in case of the occurrence of an observed HazMat accident. The Short-Cut Method (SCM) proposed in this study provides a quick and simplified method of DGT safety analysis, based on the application of the characteristic scheme of consequence analysis. Starting from the typical data of the considered transport and the most representative weather and climate conditions, the estimated damage distance—according to the data considered—for hazardous substances allows for quick hazard maps to be created for planning and emergency response procedures.

The case studies conducted in Genoa and Sardinia, which involved transport accidents with chlorine and hydrochloric acid, respectively, provide practical examples of the application of the SCM and underscore its significance. The SCM considers two categories of hazardous substances of toxic gases, for which the Short-Cut Method can provide direct results in the case of hydrochloric acid substances and the Paradigm process can provide results through its application for the toxic gas class (chlorine).

The case studies of transport accidents involving chlorine and hydrochloric acid have demonstrated the effectiveness of the SCM in providing rapid visualization of the potential outcomes of an event, allowing for quick responses to be implemented to manage the response phase.

The application of this SCM provides a rapid visualization of the potential outcome of an event by drawing the impact area for different accident scenarios, which can provide quick hazard maps for updating plans emergency procedures. Therefore, this can properly support authorities and decision-makers in monitoring and assessing potential consequences when taking actions in prevention and management of the response phase in the case of an accident involving dangerous goods for which territorial operators’ and authorities’ procedures-management-chains are trained.

## Abbreviations

ADR: Agreement concerning the International Carriage of Dangerous Goods by Road
ATB/FC: A tanker truck (ATB) or a rail tanker (FC)
CAS Number: is a unique identification number assigned by the Chemical Abstracts Service (CAS), U.S.A., to every chemical substance described in the open scientific literature
DGT: Dangerous Goods Transportation
DIPPR: Design Institute for Physical Properties
HazMat: Hazardous Material
IDLH: Immediately Dangerous to Life or Health values
LC50: Lethal concentration 50 is the amount of a substance suspended in the air required to kill 50% of a test animal during a predetermined observation period. LC50 values are frequently used as a general indicator of a substance’s acute toxicity
LOSE+LAB: Acronyms for Living LAboratory (LAB) developed as an online platform to face the tasks related to LOgistics and SafEty of goods transport
LPHC event: Low Probability and High Consequence event
Paradigm: A reference model, a term of comparison. The word comes from the Greek παράδειγμα, composed of παρα ≪between≫ and δείκνυμι ≪to show≫
SCM: Short-Cut Method
VCE: Vapor Cloud Explosion
